# The Genome Sequence of *Gossypioides kirkii* Illustrates a Descending Dysploidy in Plants

**DOI:** 10.3389/fpls.2019.01541

**Published:** 2019-11-27

**Authors:** Joshua A. Udall, Evan Long, Thiruvarangan Ramaraj, Justin L. Conover, Daojun Yuan, Corrinne E. Grover, Lei Gong, Mark A. Arick, Rick E. Masonbrink, Daniel G. Peterson, Jonathan F. Wendel

**Affiliations:** ^1^Crop Germplasm Research, USDA, College Station, TX, United States; ^2^Plant Breeding and Genetics, Cornell University, Ithaca, NY, United States; ^3^National Center of Genome Resources, Santa Fe, NM, United States; ^4^School of Computing, DePaul University, Chicago, IL, United States; ^5^EEOB Department, Iowa State University, Ames, IA, United States; ^6^College of Plant Science and Technology, Huazhong Agricultural University, Wuhan, China; ^7^Key Laboratory of Molecular Epigenetics of the Ministry of Education, Northeast Normal University, Changchun, China; ^8^Institute for Genomics, Biocomputing & Biotechnology, Mississippi State University, Mississippi State, MS, United States; ^9^Genome Informatics Facility, Iowa State University, Ames, IA, United States

**Keywords:** speciation, chromosome evolution, cotton, structural rearrangements, *Gossypieae*

## Abstract

One of the extraordinary aspects of plant genome evolution is variation in chromosome number, particularly that among closely related species. This is exemplified by the cotton genus (*Gossypium*) and its relatives, where most species and genera have a base chromosome number of 13. The two exceptions are sister genera that have n = 12 (the Hawaiian *Kokia* and the East African and Madagascan *Gossypioides*). We generated a high-quality genome sequence of *Gossypioides kirkii* (n = 12) using PacBio, Bionano, and Hi-C technologies, and compared this assembly to genome sequences of *Kokia* (n = 12) and *Gossypium* diploids (n = 13). Previous analysis demonstrated that the directionality of their reduced chromosome number was through large structural rearrangements. A series of structural rearrangements were identified comparing the *de novo G. kirkii* genome sequence to genome sequences of *Gossypium,* including chromosome fusions and inversions. Genome comparison between *G. kirkii* and *Gossypium* suggests that multiple steps are required to generate the extant structural differences.

## Introduction

One of the extraordinary aspects of plant genomes is how variable they are in terms of chromosome number. Haploid chromosome counts among angiosperms span more than two orders of magnitude, from a low of n = 2 in six different species spread among four angiosperm families (Vanzela et al., 1996; Roberto, 2005), to 320 in the genus *Sedum* (Crassulaceae) (Uhl, 1978). Driving this diversity are mechanisms that both expand and shrink chromosome numbers, either saltationally *via* polyploidy, or in a more stepwise fashion *via* ascending or descending dysploidy. These processes have long been recognized as important in speciation (Stebbins, 1971; Grant, 1981) because of the impact of chromosome number divergence on reproductive isolation. Reflective of this, it is not uncommon for congeneric species to display either ascending or descending chromosome counts. From a mechanistic perspective, ascending or descending dysploidy can arise from several chromosome rearrangement processes (Jones, 1998; Guerra, 2008; Heslop-Harrison and Schwarzacher, 2011; Lysák and Schubert, 2013; Weiss-Schneeweiss and Schneeweiss, 2013; Hoang and Schubert, 2017), including ascending dysploidy *via* chromosome fission along with the evolution of neocentromeres (Giannuzzi et al., 2013; Lysák and Schubert, 2013), and descending dysploidy through various chromosome fusion processes, including the difficult to distinguish telomere-to-telomere fusions and Robertsonian translocations (Schubert, 1992; Lysák and Schubert, 2013; Chiatante et al., 2017; Jarvis et al., 2017), and the acquisition of chromosome segments into other chromosomes (Luo et al., 2009; Murat et al., 2010; Vogel et al., 2010; Wang and Bennetzen, 2012; Fonsêca et al., 2016).

**Figure 1:**
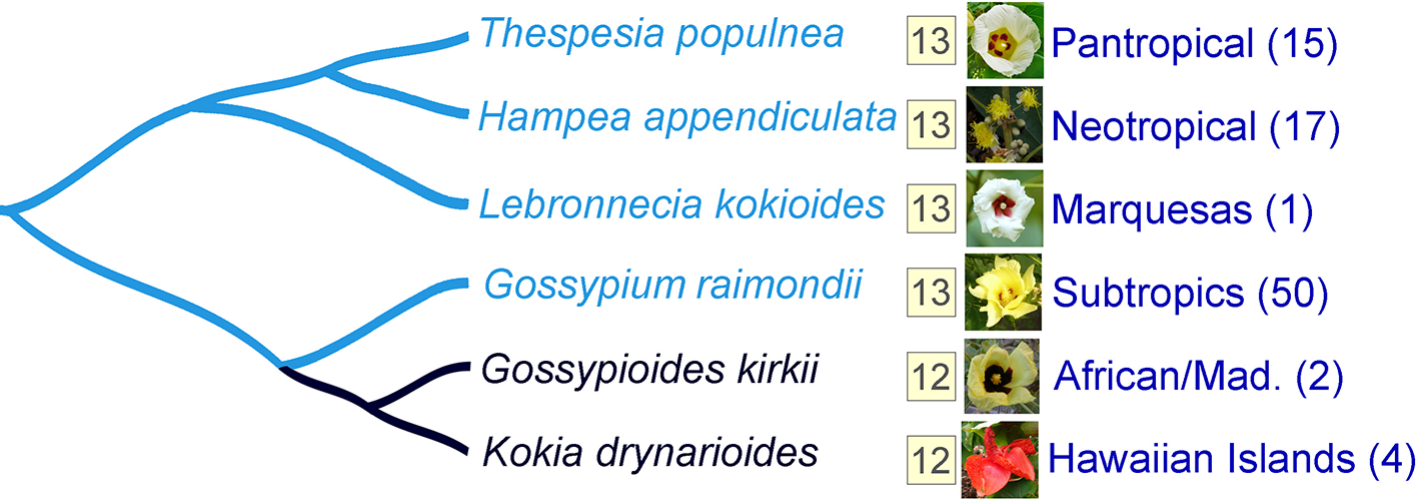
The clade containing species (*Gossypioides kirkii* and *Kokia drynarioides*) with n = 12 is nested among genera that have n = 13, suggesting that these two species have one fewer chromosome compared to their close relatives. Six different species are used as examples. The haploid chromosome number for each species and group is indicated in the yellow box. Aggregate geographic distribution (Mad. refers to Madagascar) and species richness (number of species in parentheses) are shown next to each genus. Phylogenetic tree is based off Wendel et al. (2002).

A prerequisite for understanding the directionality of chromosome number change in any taxonomic group is the availability of a well-established phylogenetic framework, so that hypotheses regarding ancestral and derived conditions are phylogenetically justified. Illustrative of this is the small monophyletic tribe *Gossypieae*, which contains the economically important cotton genus (*Gossypium*) as well as eight other lesser known genera (including *Thepparatia*) (Fryxell, 1979; Seelanan et al., 1997; Phuphathanaphong, 2006). More than 20 years ago, the Hawaiian *Kokia* and the East African/Madagascan *Gossypioides* were shown to belong to a single clade (**Figure 1**). Because these two genera have one fewer chromosomes (n = 12) than their sister genus *Gossypium* (n = 13), and because this assemblage is nested within other genera (e.g., *Hampea*, *Thespesia*) with a chromosome number of 13, they proposed an explanation involving aneuploid reduction in the lineage leading to *Kokia* and *Gossypioides* after divergence of this branch from *Gossypium*. Temporal perspectives to this reduction are the recent divergence time estimates of 5 million years (MY) for *Kokia* and *Gossypioides* and about 10 MY for the divergence of this clade from *Gossypium* (Wendel and Cronn, 2003).

Here we describe the genomic consequences of descending dysploidy in the *Kokia/Gossypioides* clade. We present a high quality *de novo* genome assembly for *Gossypioides kirkii* and compare this assembly to *Gossypium*, for which multiple assemblies have been generated. Comparison of our high quality genome assembly to other *Gossypium* genomes suggests that aneuploid reduction was accompanied by chromosome fusion and other structural rearrangements. Assuming the *Gossypium* genome was representative of the ancestral genome, we developed a model of aneuploid reduction that included several structural rearrangements reducing three chromosomes to two chromosomes during the evolution of the ancestor to the *Kokia* and *Gossypioides* genera.

## Materials and Methods

### Plant Material, Sequencing, and Assembly

*G. kirkii* leaves were collected from the Pohl Conservatory at Iowa State University and shipped to Brigham Young University for DNA extraction. Seven PacBio cells were sequenced at BYU from two libraries created from the same DNA source. Sequence reads were assembled (**Table S1**) using Canu V1.6 (Koren et al., 2017).

Leaf tissue of *G. kirkii* was also shipped to Phase Genomics (Seattle, WA) for DNA extraction and construction of HiC sequencing libraries. The sequenced HiC libraries generated 47× coverage of 125 bp paired-end Illumina reads; these were used to establish connections between contigs (**Table S2**). Illumina reads were mapped to the reference genome and a proximity guided assembly (PGA) was performed by Phase Genomics. High-molecular weight (HMW) DNA was extracted and labeled following the Bionano Plant protocol for the Irys system.

The optical map was aligned to the PGA assembly using an *in silico* labeled reference sequence. Conflicts between the Bionano map and the PGA assembly were manually identified in the Bionano Access software by comparing the mapped Bionano contigs and reference sequence to a bed file containing sequence contigs. These inter-species alignments, along with the Bionano alignments, guided manual rearrangements of scaffolds. Corrections to the PGA assembly removed conflicts between datasets by repositioning and reorienting sequence contigs in PGA ordering files. Corrections to the HiC scaffolding were made if more than one other genome agreed with the rearrangement and if the rearrangements coincided with contig breakpoints (i.e. scaffolding rearrangements). The contig order was arranged to maximize the frequency of close linkages throughout the genome. The resulting fasta file of the scaffolded assembly was produced by concatenating PacBio contigs with 100 N bases between them. Several iterations of correction and realignment resolved nearly all of the conflicts between the sequence and Bionano assemblies. Similar iterations of HiC interaction maps were created using Juicer v1.5 and Juicebox v1.8.8, respectively, for the final manual adjustments to the genome sequence. Specifically, HiC reads were re-mapped to the modified sequence and the association frequency between each paired-end was used to adjust the genome sequence using JuiceBox (Durand et al., 2016). A custom python script from Phase Genomics was used to adjust the initially assembled pseudomolecules with the changes made to the genome *via* JuiceBox. Based on the HiC data, the *G. kirkii* pseudomolecule corrections consisted of two inversions and three translocations (involving seven of 12 chromosomes). These corrections established near-complete congruence between mapped paired-ends along the entire genome. The *G. kirkii* genome sequence is available from GenBank (Accession numbers: CP032244–CP032255).

### Genome Alignments

The *G. kirkii* assembly was separately aligned by Minimap2 (Li, 2018) to other genomes in *Gossypium*, including *G. arboreum* (Du et al., 2018), *G. raimondii* (Paterson et al., 2012), and *G. hirsutum* (Zhang et al., 2015), and visualized using dotPlotly (Poorten, 2018). The alignments identified assembly errors in chromosomes Chr09 and Chr12 of *G. raimondii*. Telomere sequences were also used to confirm assembly completeness and structure by searching for the canonical telomere repeat (McKnight et al., 1997; Fajkus et al., 2005; Watson and Riha, 2010) in the *G. kirkii* genome using Geneious (Biomatters, New Zealand). The telomere repeat were also visualized and manually annotated in Geneious to verify telomere location on each chromosome.

Phase Genomics also constructed and sequenced a Hi-C library made from leaf tissue of *Kokia drynarioides*, a member of the genus sister to *Gossypioides* that also shares n = 12, to further verify the structure. The Hi-C reads were mapped to the final, corrected version of the *G. kirkii* genome assembly using BWA. Approximately 10.7 M contacts (11% of the total paired reads) passed mapping filters and were used as Hi-C interaction evidence. Contact maps were visualized using Juicer v1.5 and Juicebox v1.8.8.

### Transcriptomic Sequencing and Gene Annotation

Total RNA was extracted from 3-cm seedling leaves. Illumina TruSeq RNA-sequencing libraries were prepared for each replicate and were sequenced (Paired-end 150 bp) Berry Genomics Co. Ltd. (Beijing, China). Gene annotations were created using GenSAS 5.0 (Lee et al., 2011), an online integrated genome sequence annotation pipeline. BUSCO analysis was conducted to test for annotation completeness. Repetitive elements were detected by RepeatModeler (Smit and Hubley, 2013) and RepeatMasker (Smit et al., 2019). AgriGO tested for enrichment of Gene Ontology categories of gene functions in the rearranged segments (Tian et al., 2017). RNA-seq of *G. kirkii* is available from GenBank under SRX5894875. Gene and repetitive annotations are available from CottonGen under https://www.cottongen.org/analysis/213.

### Analysis of Paleo-Genome Duplications

Protein sequences of *G. kirkii* and the D_T_ genome of *G. hirsutum* were clustered using OrthoFinder v.2.1 (Emms and Kelly, 2015) with the Diamond alignment tool. Single copy orthologs from OrthoFinder were used as input to MCScanX_h (.homology file), with default settings (Wang et al., 2012). The collinearity plots between chromosomes Chr02 (Chr15, if the tetraploid chromosomes were numbered sequentially), Chr04 (Chr17) and Chr06 (Chr19) in *G. hirsutum* and chromosomes KI_2_4 and KI_06 in *G. kirkii* were created using the circle_plotter downstream tool of the MCScanX package. From the OrthoFinder output, all of the intraspecific paralogs were extracted for *G. kirkii*. Within each group of putatively orthologous genes, Ks values for every possible pairwise combination of paralogs were calculated using the codeml package of PAML, using custom python scripts.

### ChIP-seq

Leaves and leaf buds were also collected from *G. kirkii* (specimen voucher ISC 418555, Ada Hayden Herbarium, Iowa State University). Rabbit polyclonal CenH3 antibody was made to the CenH3 amino acids 9–20 and conjugated to KLH (Covance, Inc.), a conserved peptide in *Gossypium* species of CenH3. Immunostain on *G. raimondii* root tips ensured centromere specificity of the CenH3 antibody. Chromatin immunoprecipitation was performed using the Epigentek EpiQuik Plant ChIP Kit (P-2014) with modifications. DIECA (2%) and PVP-40 (4%) were added to the fixative and to final solutions of CP3C, CP3D, and CP3E. DNA samples were sonicated at 60% amplitude for three total minutes of sonication/rest (15 s/15 s). Divided samples were incubated with either rabbit pre-immune sera, anti-CenH3, or polyclonal H3K9ac (ABCam, ab10812, LOT GR171780). Four replications of each reaction was pooled for whole genome amplification using the SeqPlex Enhanced DNA Amplification Kit (SeqXE, Sigma) then sequenced (Illumina PE150 bp) at the Beijing Genomics Institute (BGI). ChIP-seq reads were mapped to the genome using BWA (Li and Durbin, 2009). ChIP-seq data are available from NCBI under SRX5894872–SRX5894874.

### FISH

Preparation of chromosomes and staining were performed as previously described for maize (Masonbrink and Birchler, 2010). FISH was performed as specifically described for cotton (Wang et al., 2006).

## Results

### Sequencing and *De Novo* Assembly of the *G. kirkii* Genome

**Figure 2:**
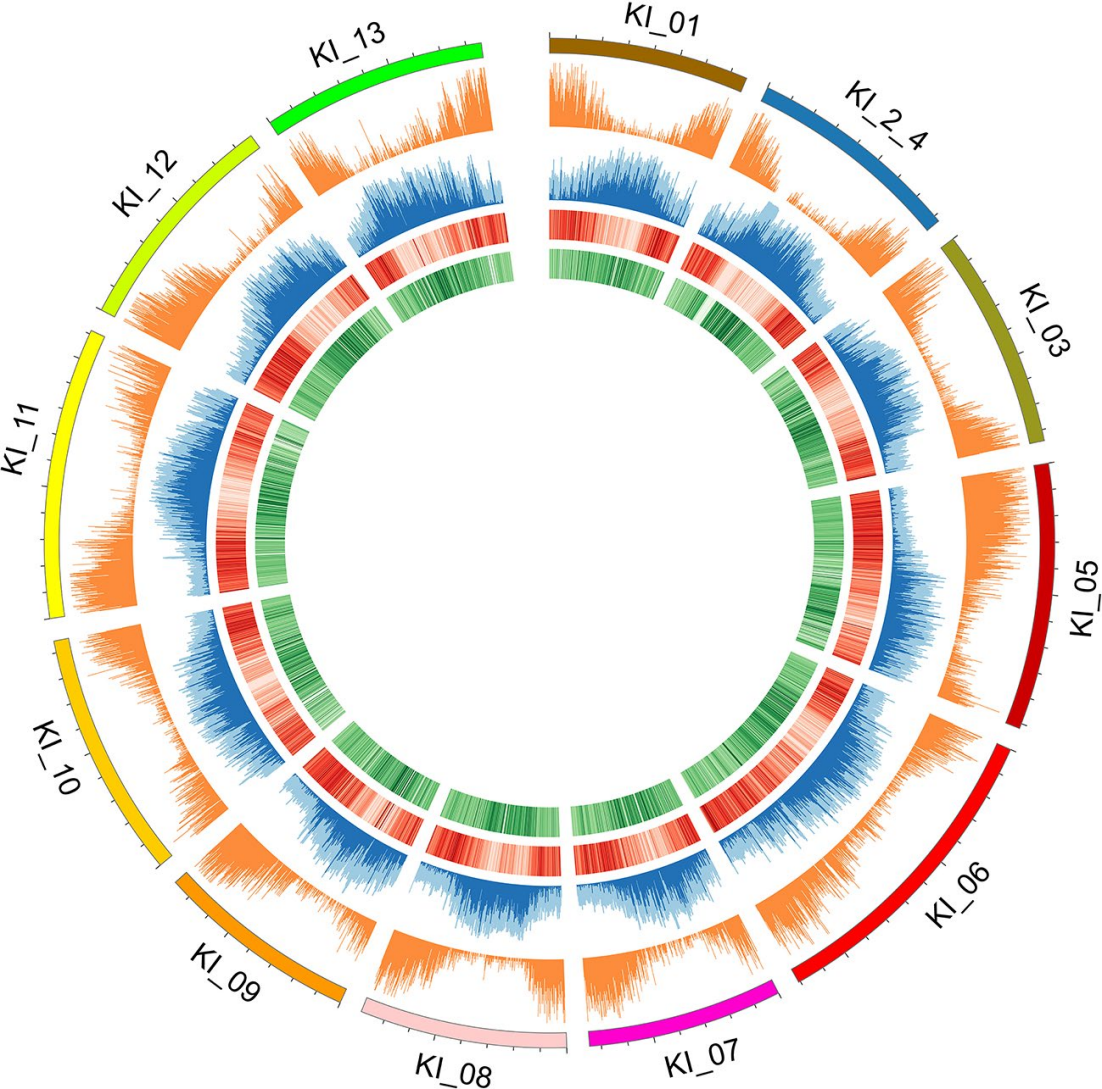
Individual chromosomes (KI_ labels) of the *G. kirkii* genome are illustrated by 5 tracks in a Circos plot. Darker shades of colors represent a higher value or frequency of genomic features with the 100 kb window. From outside to inside: chromosome graph and scale; plot of gene density; TE content (light blue is total TE content, dark blue is Gypsy content), ChIP-seq of H3K9ac (Darker red lines of H3K9ac indicate a higher frequency of H3K9 acetylation); and ChIP-seq of CENH3 (Darker green lines of the CENH3 track indicate a higher frequency of CENH3 binding). Centromeres are inferred in the regions where H3K9ac is low (light red) and CENH3 is high (dark green).

Two different genome technologies were used to assemble the *G. kirkii* genome sequence (**Figure 1**). First, approximately 68× coverage of raw SMRT data (40 Gb) was generated using the PacBio Sequel System (**Table S1**). The contig-level assembly was 544 Mb composed of 389 contigs with a contig N50 of 9.92 Mb and a maximum contig size of 31.1 Mb (**Table 1**). After scaffolding with HiC (Burton et al., 2013), the 12 pseudomolecules assembly was 92.5% of the expected genome size of 588 Mb (Wendel et al., 2002) with only 277 gaps (**Table 1**, **Table S2** and **Figure S1**). Chromosomes were manually adjusted (**Figures S2** and **S3**) and named according to the convention used in *Gossypium hirsutum* (Zhang et al., 2015). These pseudomolecules represented the 12 chromosomes of the *G. kirkii* genome (**Figure 2**, **Table S3**). Chromosome KI_2_4 contained the largest number of sequence contigs (65 contigs, 41.5 Mb) and Chromosome KI_08 contained the fewest (seven contigs, 39.6 Mb), even though these two chromosomes contained approximately the same total sequence length. Chromosome KI_06 was the largest chromosome (see below).

**Table 1:** Assembly metrics of the *Gossypioides kirkii* genome.

Genome Statistics	Stats for Contigs	Stats for Pseudomolecules (Hi-C)	Stats for Chromosome Assembly (BioNano)
*Assembly size (in Mb)*	544	538	354
*Number of sequences (number of gaps)*	389 (0)	12 (277)	1,079 (3,721)
*Longest Scaffold Length (Mb)*	31.1	60.3	16.7
*N50 in Mb (mean number of contigs per chromosome)*	9.92 (17)	42.97 (17)	0.296

An optical map (Bionano Genomics, Inc.) was used to validate the assembly of individual contigs and the HiC connections between contigs (**Table S4**). Optical map data typically serves as an independent validation of the assembled sequence because the image data of Bionano labeled DNA molecules is assembled independently and aligned to DNA sequences using restriction patterns matching the labels in the Bionano contigs (Udall and Dawe, 2017). While the percentage of alignments between optical maps and contigs was relatively low, we note that over half of the genome sequence was validated by optical map alignment. The Bionano alignments also spanned 62% of the 71 eligible sequence gaps (i.e. gaps flanked by contigs >100 kb on each side, since Bionano contigs do not generally match smaller contigs due to limitations in nick-pattern matching) (**Dataset S1**).

A common measure of genome quality is the percentage of expected genes recovered in an annotated assembly. Here, the percentage of genes identified in the *G. kirkii* genome sequence provided confidence that nearly the entire genome was represented, with 95% (1,364/1,440) of conserved genes from BUSCO identified (Simão et al., 2015). The remaining genes were either fragmented (n = 18, 1%) or missing (n = 58, 4%). That a few of the BUSCO genes were missing is not surprising due to the previously reported genome downsizing and gene loss in this species (Grover et al., 2017), and therefore may reflect a combination of genome completeness as well as historical evolution. Of the 36,669 gene annotations, 64% had RNA-seq reads (> 20 reads) mapping to them, suggesting that we assembled much of the leaf transcriptome.

### Collinearity With Other *Gossypieae* Genomes

The integrity of the *G. kirkii* genome assembly was also assessed by comparing it to genome sequences recently published for *Gossypium* (Paterson et al., 2012; Du et al., 2018; Wang et al., 2019) (**Figures S4**–**S7**). Occasionally, we used these comparisons to correct scaffolding errors in the *G. kirkii* genome if the *G. kirkii* contigs and optical map contigs supported such corrections. These manual rearrangements (**Dataset S2**) utilized evidence from both contig ends of each initial non-colinear placement of *G. kirkii* sequence.

We further assessed the completeness of the assembly and the orientation of terminal scaffolds by searching for telomere sequences in the *G. kirkii* pseudomolecules. We identified 20 loci with characteristic sequence of telomere repeats at the ends of our pseudomolecules (**Table S5**); eight pseudomolecules had telomere repeats on both chromosome arms, four had telomere repeats on a single arm, and two pseudomolecules had telomere repeats that were confidently embedded within a single scaffold. The longest telomeric repeat (> 24 kb) was identified on KI_04. Since this length was longer than most of the length of our trimmed reads used for assembly (N50 = 16,192), it is likely that many reads containing a majority of telomeric sequence collapsed during sequence assembly. Indeed, these regions had a higher read coverage compared to the adjacent chromosome sequence (data not shown). Different telomere sequences were identified in different combinations on each of the chromosome ends, suggesting the existence of multiple telomerases or at a minimum multiple guide RNAs in *Gossypioides*.

Because typical centromeres do not have conserved sequences (Ma et al., 2007; Lysak, 2014; Birchler and Han, 2018), we leveraged additional data to identify centromeric regions. That is, we evaluated the density of both ChIP-seq reads and gene density to infer putative centromeric regions. Euchromatic and histone modifications of H3K9ac and CENH3, respectively were used to estimate centromeric regions (Masonbrink et al., 2014). Typical distributions of epigenetic marks were identified (e.g. increasing frequency of CENH3 marks near the centromeric regions, **Figure 2**). In some cases, chromosomes had a single contig assembled across the centromeric region (e.g. chromosomes KI_06, KI_10, KI_11) suggesting proper assembly and density of CENH3 marks in centromeric regions. The centromeric regions of other chromosomes contained multiple contigs. While their assembly depended on both correct sequence assembly and correct scaffolding, their density of CENH3 marks was similar to those regions composed of a single contig.

### Annotation of Genes and Repetitive Elements

Gene annotation recognized 36,669 genes, somewhat higher than previously reported (Grover et al., 2017); these differences are likely due to both genome quality and annotation method. All *G. kirkii* genes were aligned to their closest intragenomic paralog to calculate synonymous substitutions (Ks); the plot of these pairwise Ks values exhibits a peak congruent with previous findings (Conover et al., 2019) of an ancient polyploidization event shared with *K. drynarioides* and all members of *Gossypium* (**Figure S8**). Because genes comprise useful genomic anchors, gene annotations were used to inform analyses of the chromosome rearrangements in *G. kirkii* (below).

Repetitive elements were detected by RepeatModeler (Smit and Hubley, 2018) and RepeatMasker (Smit et al., 2019). As a whole, the genome contained ∼30% interspersed repeats and 1.7% simple repeats. The interspersed repetitive elements corresponded to transposable elements, namely *Gypsy* and *Copia* retrotransposons (**Table S3**). We detected the TEs on each chromosome to assess the class distribution of TE elements throughout the genome (Bailly-Bechet et al., 2014). While TEs can be associated with chromosome rearrangements, we found no bias in terms of TE number, total length, or class between chromosomes. In general, the number and total length of *Gypsy* elements greatly outweighs *Copia* elements, as is common for many plant genomes (**Table S3**).

### Comparative Genomics Between *G. Kirkii*and Related Genomes

**Figure 3:**
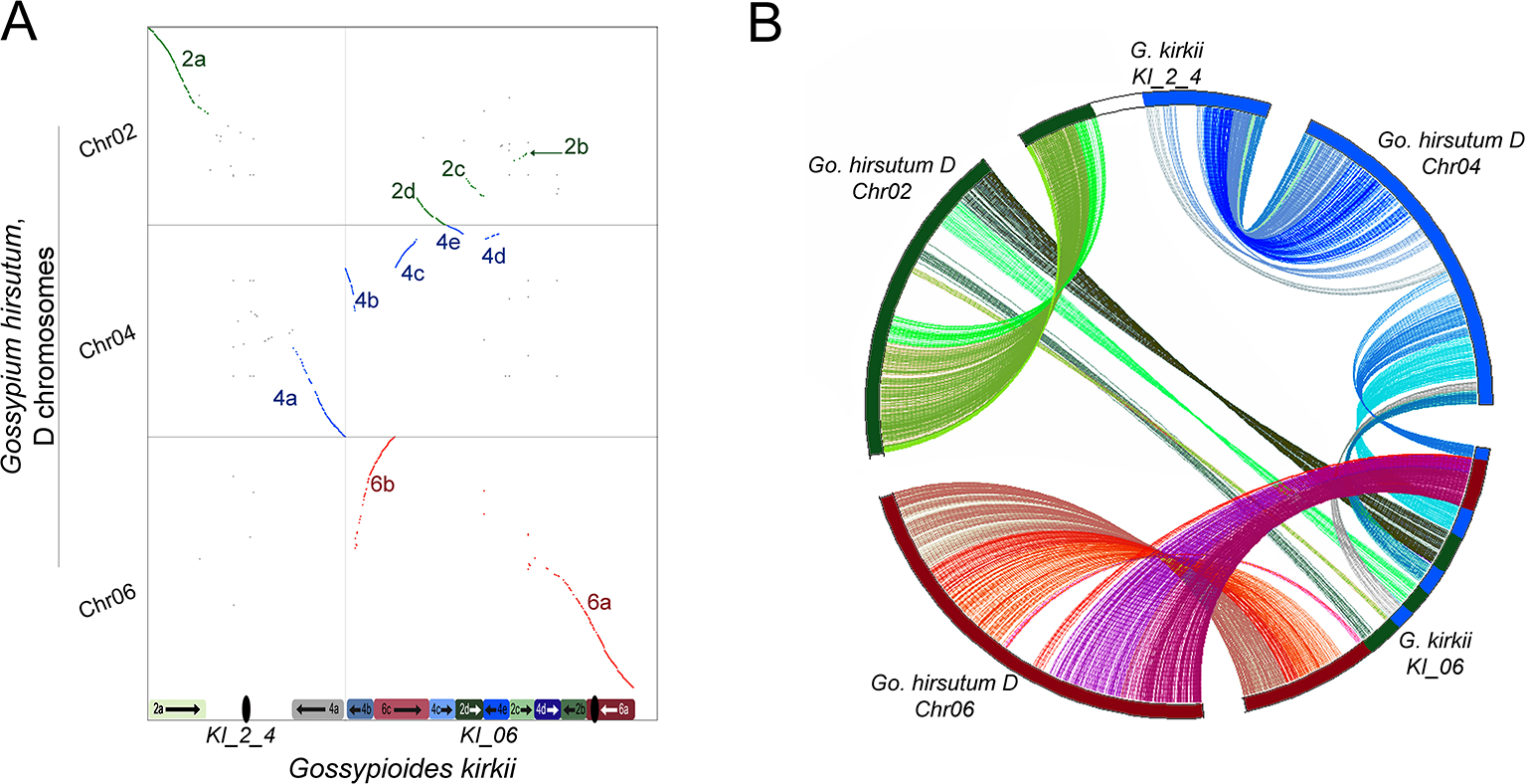
**(A)** Whole-genome dot-plot alignment between the aneuploid-reduced chromosomes of *Gossypioides kirkii* (Chr2_4 and Chr06; x-axis) and the ancestral state, represented here by the D_T_-genome of *Gossypium hirsutum* (Chr02, Chr04, Chr06; y-axis) because of an assembly error in *Gossypium raimondii* affecting a key chromosome. Diagrams of the current chromosome configurations are represented next to each of their respective axis **(B)** Circos plot illustrating the rearrangement based on conserved single copy orthologs. Colors in each plot were produced to illustrate matching segments between the whole-genome and gene-based illustrations.

The base chromosome number (x) of *G. kirkii* and *K. drynarioides* is x = 12, but the remainder of the cotton tribe (*Gossypieae*) in which this lineage is nested has a base chromosome number of x = 13 (**Figure 1**). To explore which chromosomes may be involved in this derived state, we identified chromosome rearrangements that occurred after divergence between *G. kirkii* and *Gossypium* (represented by the ancestral “G” chromosomes). In this analysis, the genome of *Gossypium* was assumed to represent the ancestral genome to the *Gossypium*–*Gossypioides*–*Kokia clade*, and the *G. kirkii* genome was considered derived due to the presence of necessary changes during chromosome reduction, although we cannot discount the possibility of some structural changes in *Gossypium*. Whole genome comparisons suggested that an entire arm of chromosome G2 and an entire arm of chromosome G4 (intact within modern-day *G. raimondii* and *G. arboreum*) fused to form a single chromosome, while the other chromosome arms were fragmented and inserted into KI_06 (**Figure 3A**). A comparison of annotated genes to *G. hirsutum* in these regions also supports our inferred genome alignments (**Figure 3B**). The insertion of these G2 and G4 fragments into KI_06 explain the absence of a single chromosome that is twice the size of other metacentric chromosomes, as might be expected if a simple chromosome fusion had occurred (Hutchinson, 1943). We confirmed the absence of an unusually long chromosome in *G. kirkii* by chromosome staining (**Figure S9**). The inserted portion on KI_06 consists of alternating segments of ancient chromosomes G2 and G4 with six segments accounting for approximately 30 MB of the chromosome. Details of the rearranged segments are found in **Table S6**. These segments each contained between 117 and 458 genes. A GO enrichment test of each segment found no enriched GO categories.

These findings may be summarized as three salient facts regarding the genomic history of *G. kirkii*. First, one chromosome arm each of G2 and G4 were inserted into what became part of *Gossypioides kirkii* chromosome KI_06. Second, before or after the insertion, segments of these two chromosome arms were interleaved through unknown evolutionary processes. It is worth noting that all of the G2/G4 ‘junctions’ in KI_06 have strong support of PacBio and Bionano coverage. Third, the remaining, entire chromosome arms of G2 and G4 fused to create KI_2_4. We further support these inferences by mapping a *K. drynarioides* HiC library to the *G. kirkii* assembly. The resulting HiC contact heatmap (**Figure S10**) also shows a linear contact pattern along KI_06 and KI_2_4 suggesting that the chromosome rearrangements we describe are shared between these sister genera *Kokia* and *Gossypioides*.

## Discussion

Among the many opportunities afforded by genome sequencing is the possibility of gaining insight into long-standing cytogenetic phenomena that remain unexplained at the sequence level. A promising example is dysploid evolution, which is a well-known and common pattern of cytogenetic variation in both plants and animals (Grant, 1971; Stebbins, 1971; White, 1973). From a mechanistic standpoint, it has long been thought that dysploidy arises primarily from chromosome translocations. This view was promulgated in George Ledyard Stebbins’ 1971 classic *Chromosomal Evolution in Higher Plants*, in which he stated that “*aneuploid alterations of the basic chromosome number are usually the outcome of successive translocations*” (5, pg. 86). Similarly, in Verne Grant’s widely used 1971 textbook “*Plant Speciation*”, he stated “*the mechanism of aneuploid reduction at the diploid level involves unequal reciprocal translocations*” (4, pg. 359). More recently, telomeric (end-to-end) fusion and Robertsonian translocation have been recognized as processes leading to aneuploid reduction (Schubert, 1992; Lysák and Schubert, 2013; Chiatante et al., 2017; Jarvis et al., 2017), as has the insertion of one chromosome into another (Luo et al., 2009; Murat et al., 2010; Vogel et al., 2010; Wang and Bennetzen, 2012; Fonsêca et al., 2016). Remarkably, while we were completing the present work, Birchler and Han (Birchler and Han, 2018) published a thought-provoking explication of how the Breakage-Fusion-Bridge cycle, as illuminated by McClintock 80 years ago for understanding various chromosome anomalies in maize (McClintock, 1939; McClintock, 1941), likely has causal connections to common mechanisms of karyotypic evolution in plants, and by extension possibly all eukaryotes.

Here we provide sequence-based evidence for chromosome number reduction where related members of the cotton tribe establish the polarity of the descending dysploidy (from x = 13 to x = 12). The foundation for our conclusions is the high-quality assembly of the *G. kirkii* genome sequence presented here. The accuracy of this assembly was determined by multiple congruent datasets (PacBio, HiC, and Bionano) and by comparative analyses that demonstrate consistency with previously published cotton genomes (Paterson et al., 2012; Zhang et al., 2015; Du et al., 2018; Wang et al., 2019). Analyses of colinearity revealed a complex pattern of inter-digitating chromosome segments. The identified rearrangements also are congruent with previous cytogenetic observations of *Gossypioides brevilanatum*, in the fact that *G. kirkii* does not display an ‘extra-large’ chromosome (Hutchinson, 1943) as might be expected from a simpler scenario of a 2 to 1 chromosome fusion event.

Explanations for the inferences depicted in **Figure 3** for the derivation of the reduced chromosome number in *G. kirkii* (“KI” chromosomes) relative to the G chromosomes of ancestral *Gossypieae* need to account for the following observations: (1) identification of end-to-end G2 and G4 (2d and 4e) segments (including internal telomeric sequences) in KI_06 that implicate a historical end-to-end fusion of ancestral chromosome arms G2 and G4 (2) chromosome KI_2_4 contains entire chromosome arms of G2 and G4, suggestive of chromosome fusion at, or close to, the centromeres for the intact G2 and G4 arms; and (3) because the terminal inversion on KI_06 included 2.0Mb of the G4 chromosome (in addition to 8.4 Mb of the original G6 chromosome arm), it must have occurred after the insertion event above.

We recognize that by assuming the *Gossypium* genome represented the ancestral *Gossypieae* genome, unique *Gossypium* changes were confounded with the differences between *G. kirkii* and *Gossypieae*. We are comfortable with this assumption based on previous cytological work that prompted the previous generation of botanists to coin the phrase ‘cryptic structural differentiation’ when working with the cotton tribe (Fryxell, 1979). They understood the chromosomes were different based on pairing data, but observable structural differentiation was not sufficient to differentiate members of the cotton tribe, other than the descending dysploidy of *Gossypioides* and *Kokia*.

**Figure 4:**
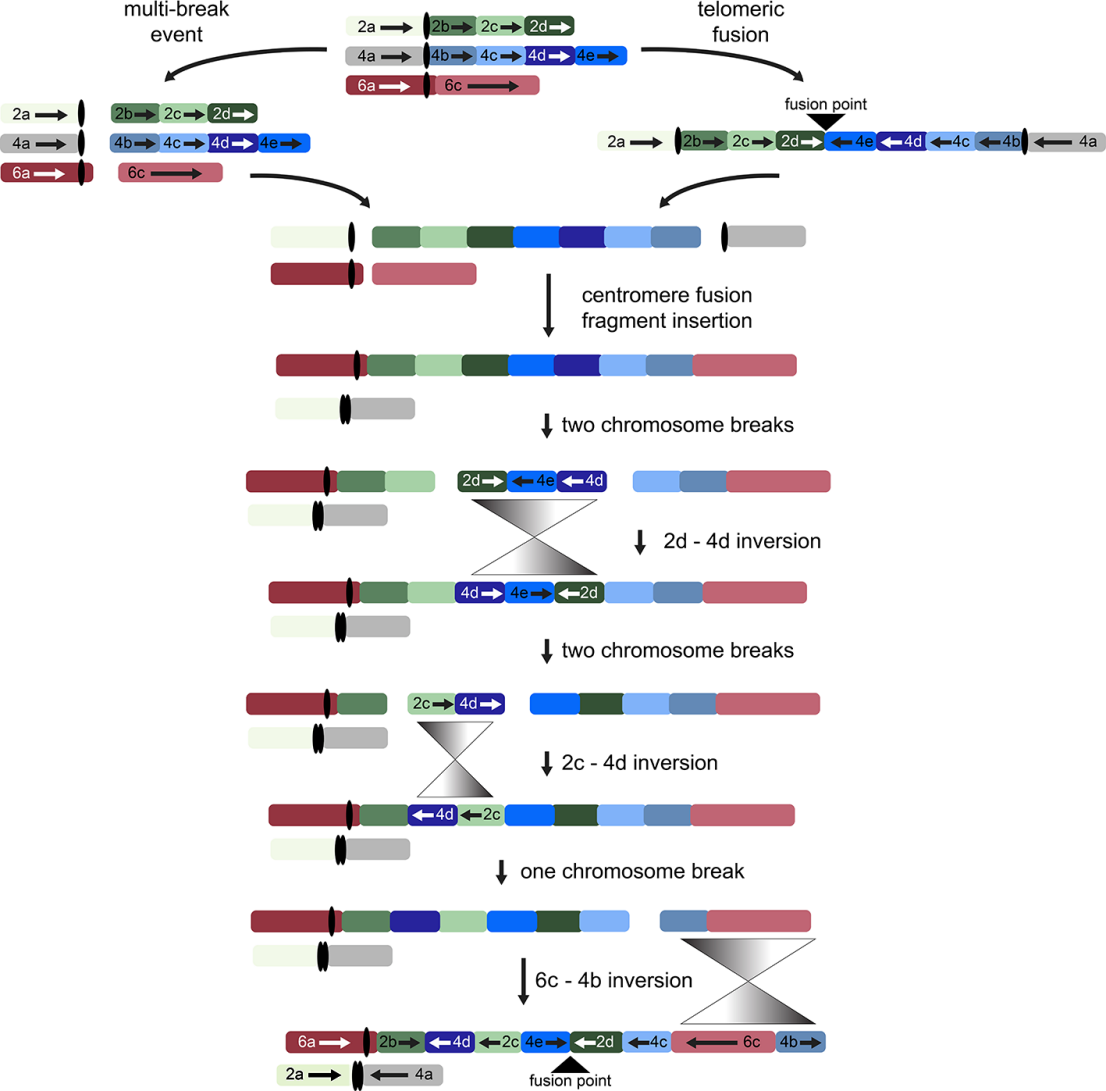
Possible evolutionary model for the origin of descending dysploidy in the ancestor of *Gossypioides* and *Kokia* (x = 12) from a progenitor with n = 13. Ancestral chromosomes involved in the aneuploid reduction are pictured at the top. Two possible paths are shown, which include a multi-break event near the centromeres of each ancestral chromosome (left) or an end-to-end fusion of ancestral chromosomes G2 and G4 (right). After either a multi-break event (left) or the generation and subsequent breakage of a dicentric chromosome, chromosome segments 2a and 4a fused to generate one chromosome, while the remaining fragments of G2 and G4 were inserted between segments of G6 (here, 6a and 6c). Three inversions (grey triangles) are required to rearrange the order of the original chromosome blocks into the pattern seen in the extant *G. kirkii* genome.

While other interpretations could be made when additional genomes from tribe *Gossypieae* (e.g., *Thespesia*, *Hampea*, or *Lebronnecia*) are sequenced, we use the above three key observations (and one modest assumption) to create a hypothesis for the order of events following initial dicentric chromosome formation (**Figure 4**). The end-to-end fusion of G2 and G4 strongly supports evolutionary models that begin with a dicentric chromosome, although we note that a multi-break-fusion event could bypass the need for a dicentric chromosome (as noted in **Figure 4**). Myriad alternative models are also possible where end-to-end fusion are coincidental instead of contributional; however, they are not considered further because of the key evidence of the telomeric sequence and directionality of the end-to-end fusion fragments.

Chromosome comparisons between *Gossypioides* and *Gossypium* suggest that the origin of *G. kirkii* KI_06 involved both fusion and a series of inversions to generate the observed interleaved pattern (**Figure 4**). While this fusion could have been the result of breaks occurring on each of the involved chromosomes, the presence of internal telomeres supports an end-to-end fusion, generating a dicentric chromosome. Perhaps, the nascent dicentric chromosome somehow contributed to Subsequent breaks near each centromere (G2- and G4-derived). If an additional break was concurrent in G6, then translocations followed by subsequent paracentric inversions could create the extant chromosomes of *Gossypioides*. As depicted in **Figure 4**, two-breaks of a dicentric chromosome created an acentric fragment containing most of the arms of G2 and G4, which inserted into G6, and centromeric fusion between the G2- and G4-chromosome arms containing centromeric sequence. Three inversions are then required to transform the initial fusion of *G. kirkii* Chr06 into the extant chromosome morphology. The two unshared inversions would only involve portions of the inserted segment. Notably, the two chromosome inversions of *G. kirkii* were approximately 9.6 Mb and 12.7 Mb, respectively (**Table S6**), which is similar to the average inversion size for plants and animals (i.e., 8.4 Mb, (Wellenreuther and Bernatchez, 2018)). While inversions often are associated with TEs (Kidwell and Lisch, 2000), we do not find an increased density of TEs in KI_06 (**Figure 2**) to support this for *G. kirkii*. Although the responsible inversion mechanism is not known, it is possible that recombination between a hemizygous insertion KI_06 and a normal KI_06 could have played a role.

The foregoing hypothesis explains a novel “3” to “2” route for chromosome number reduction, as opposed to the more conventional “2” to “1”. It certainly invokes a series of seemingly unlikely events, including formation of multiple inversions requiring two simultaneous double-strand breaks and repair (Kirkpatrick, 2010), either through a known mechanism such as breakage-fusion-bridge or unknown accidents of aberrant recombination (as described here). Because the likelihood of each rare event multiplies when each is considered as independent of the others, perhaps it is more parsimonious to postulate that chromosome number reduction occurred within a single generation, in a series of germ-line cell divisions with subsequent ‘healing’ in the sporophyte. In contrast, it remains possible that this entire process unfolded in a stepwise fashion during long evolutionary timescales. Unfortunately, we lack surviving intermediates that might testify to this temporal possibility, and we are unaware of other methods that might be used to distinguish between the “fast” and “slow” scenarios. Several other studies have detected aneuploid reduction between related species in the Brassicaceae (Lysak et al., 2006), or in the genomes of grasses (Zhang et al., 2008; Murat et al., 2010; Wang et al., 2015; Luo et al., 2017), based on patterns of FISH or using sequence comparisons, and others have noted instances of “chromosome shattering” with possible mechanisms (Zhang et al., 2008; Tan et al., 2015; Mandáková et al., 2019). As more plant and animal genomes are sequenced and assembled by robust methods, the spectrum of causative mechanisms and their frequency in explaining patterns of karyotypic evolution are likely to become much clearer.

## Data Availability Statement

The *G. kirkii* genome sequence is available on GenBank (Accession numbers: CP032244-CP032255).

## Author Contributions

JU and JW developed the idea. JU, JW, JC, and CG designed the project. EL, DY, LG, MA, RM, and DP generated the data. JU, TR, JC, DY, CG, and MA analysed the data. JU, JC, CG, and JW wrote the manuscript. All authors read, edited, and approved the final manuscript.

## Funding

Primary funding was provided by the National Science Foundation Plant Genome Research Program (#1339412). Additional support was provided from Cotton Incorporated and USDA-ARS (58-6402-1-644 and 58-6066-6-59).

## Conflict of Interest

The authors declare that the research was conducted in the absence of any commercial or financial relationships that could be construed as a potential conflict of interest.
