# Supplementary material for: The Genome Sequence of Gossypioides kirkii Illustrates a Descending Dysploidy in Plants
Source: Front Plant Sci. 2019 Nov 27;10:1541. doi: 10.3389/fpls.2019.01541 (PMC6890844; doi:10.3389/fpls.2019.01541)
Supplement: Supplementary file 3 [file Presentation_1.pdf]

Supplementary Information for

Comparative Genomics Implicates a Breakage-Fusion-Bridge Mechanism for Descending Dysploidy in Plants

Joshua A. Udall[1,*], Evan Long[3], Thiruvarangan Ramaraj[4], Justin L. Conover[2], Daojun Yuan[2], Corrinne E. Grover[2], Lei Gong[5], Mark A. Arick II[6], Rick E. Masonbrink[7], Daniel G. Peterson[6], and Jonathan F. Wendel[2,*]

[1] Crop Germplasm Research, USDA, College Station, Texas, USA
[2] EEOB Department, Iowa State University, Ames, Iowa, USA
[3] Plant Breeding and Genetics, Cornell University, Ithaca, New York, USA
[4] National Center of Genome Resources, Santa Fe, New Mexico, USA
[5] Key Laboratory of Molecular Epigenetics of the Ministry of Education, Northeast Normal University, Changchun, Jilin, China
[6] Institute for Genomics, Biocomputing & Biotechnology, Mississippi State University, Mississippi State, Mississippi, USA
[7] Genome Informatics Facility, Iowa State University, Ames, Iowa, USA

*** Correspondence:**
Josh Udall
Joshua.udall@usda.gov

Jonathan Wendel
jfw@iastate.edu

This PDF file includes:

Figs. S1 to S10
Tables S1 to S6
References for SI reference citations

Other supplementary materials for this manuscript include the following:

Dataset S1 Gaps in sequence scaffolds spanned by Bionano optical contigs
Dataset S2 Manual edits to the genome sequence in AGP file format

Fig. 1. HiC libraries were sequenced and aligned to the contigs of the PacBio assembly (white boxes). This image is a “self-alignment” of the final Phase Genomics scaffolded contig assembly of the *G. kirkii* genome sequence. The contigs of the assembly are arranged in linear order on both the x- and y-axis. The diagonal contains white boxes which represent individual contigs. Since contigs cannot be linked to itself, they appear as white boxes along the diagonal. Off the diagonal, are colored points representing log10 linkages (or proximity) of two different sequences of contigs within the nucleus. The frequency of two linked sequences as determined by sequencing the HiC library is displayed on a white-red color spectrum. The darker the color of the points, the more frequently two sequences were associated *in vivo*.

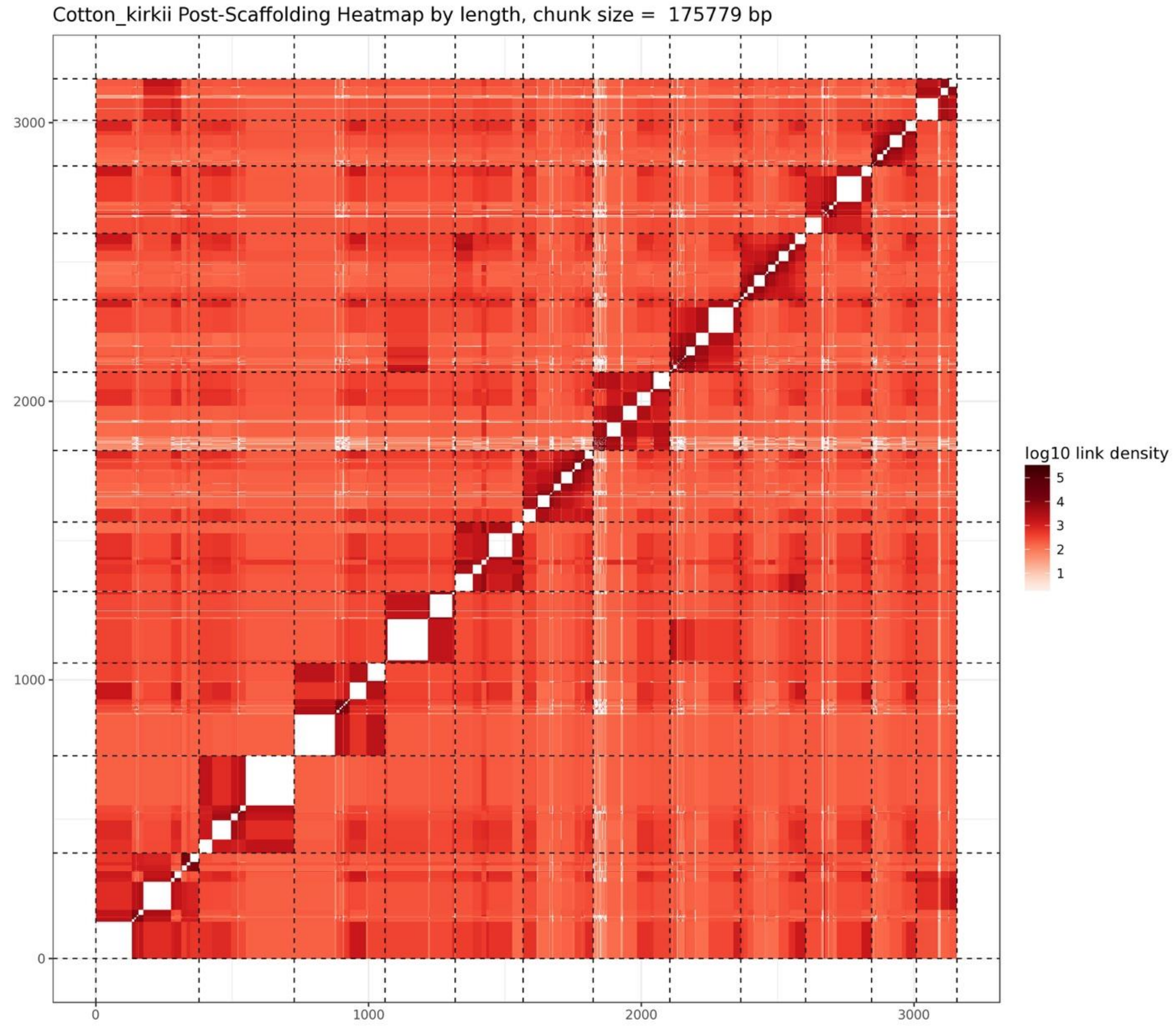

Fig. 2. Similar to Figure 1, the x- and y-axis are the scaffolds of the assembled genome sequence. The red dots represent the frequency of association (or interaction) between two points in the genome. The diagonal illustrates that most *in situ* connections of mate-pair reads are local. Thus, they form a strong diagonal. There are a few exceptions. For example, the green arrow illustrates a piece of chromosome KI_05 that has strong associations with KI_07 and suggesting that that it should be placed on KI_07. Thus, strong areas of dark red that are off diagonal between only a pair of chromosomes indicates a piece of genome that could be out of order. A final correction of the genome assembly was performed using Juicebox. There are also some red areas of a single chromosome that are shared among ALL chromosomes. In this figure, these common associations are connections between telomeres that may be positioned in close proximity in the nucleus.

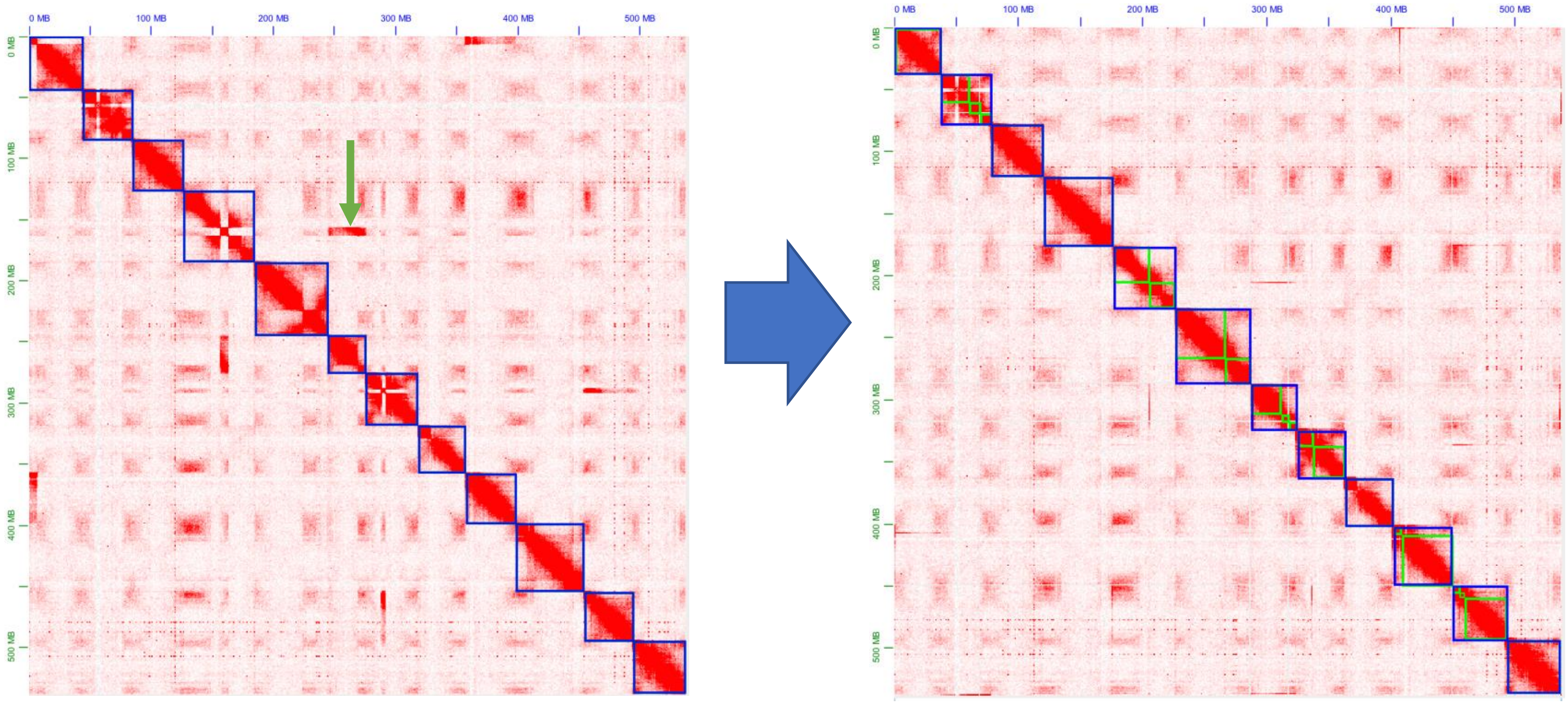

Fig. 3. A zoomed in view illustrating the manual correction to KI_05/KI_07 using Juicebox.

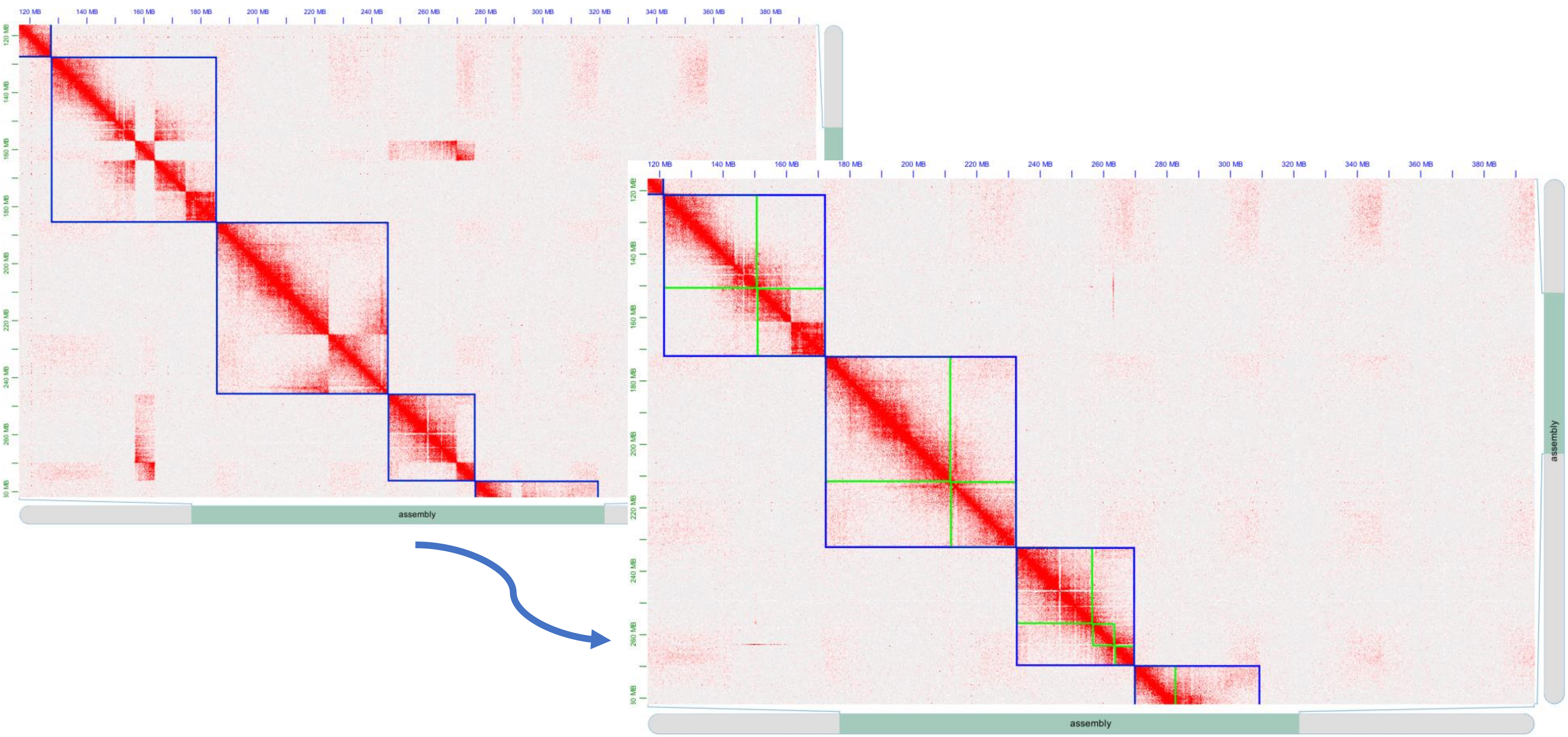

Fig 4. Dotplot of genome alignments between the genome sequence of *G. kirkii* and the genome sequence of *G. arboreum* (A2).

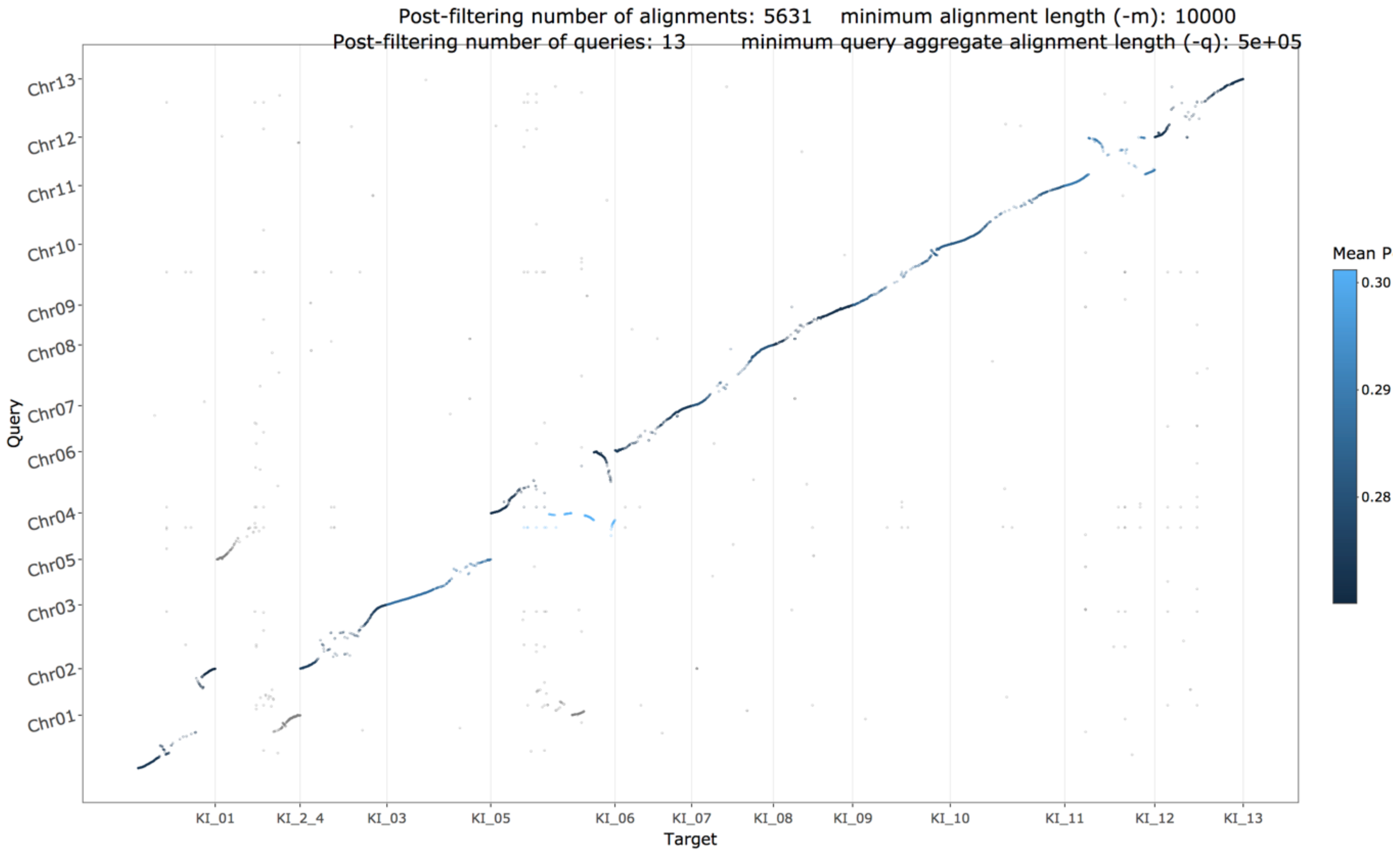

Fig 5. Dotplot of genome alignments between the genome sequence of *G. kirkii* and the genome sequence of *G. raimondii* (D5) genome. There are assembly errors in the *G. raimondii* sequence. Specifically, the errors between Chr09 and Chr12 are apparent in this dotplot. Note that the published D5 chromosome names are non-conventional. During the analysis of this body of work, we used the updated chromosome names that have been adopted by the broader research community since the publication of the tetraploid genome sequences.

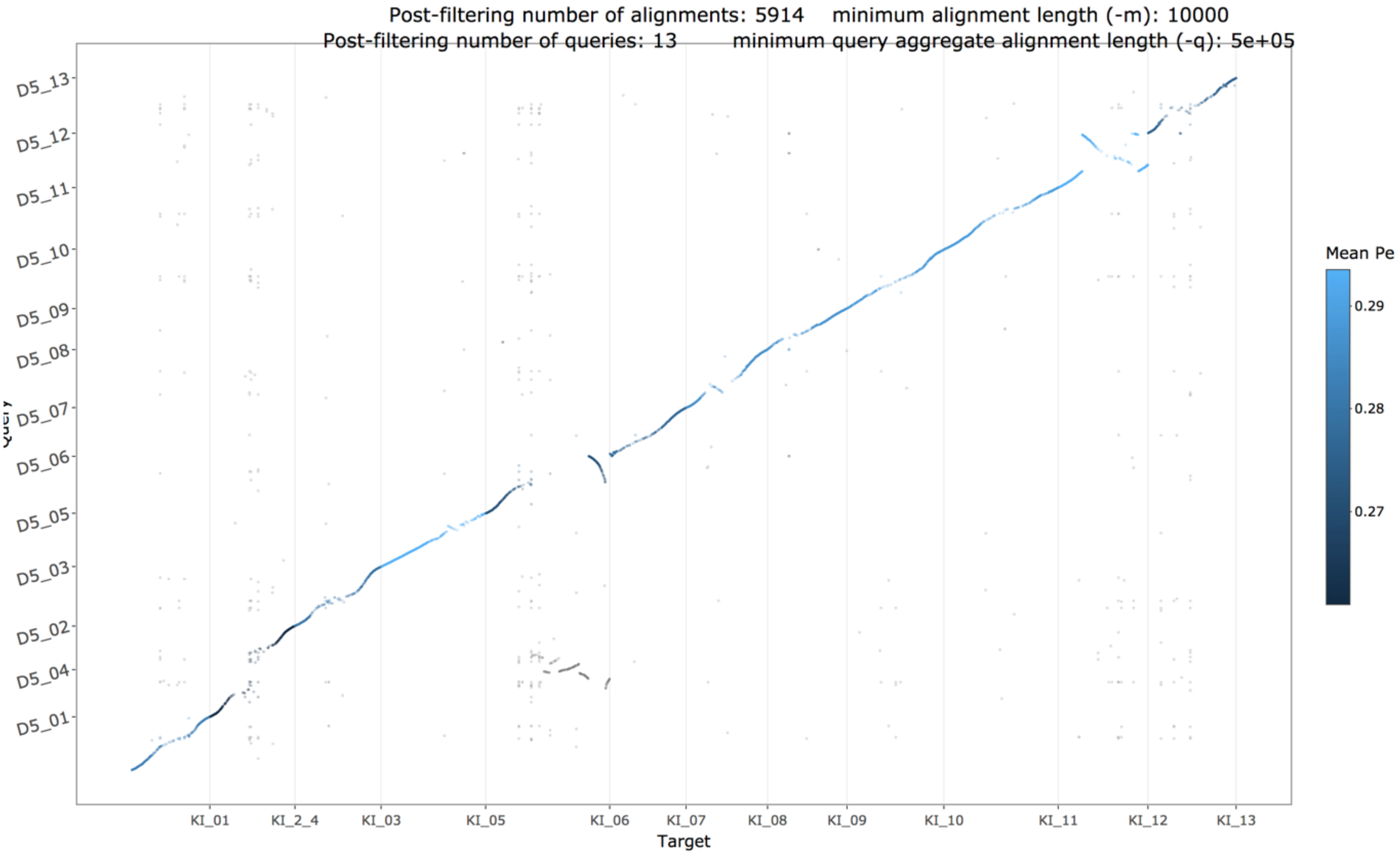

Fig 6. Dotplot of genome alignments between the genome sequence of *G. kirkii* and the genome sequence of A_T genome of *G. hirsutum* (where the 'T' indicates the A genome in the tetraploid nucleus).

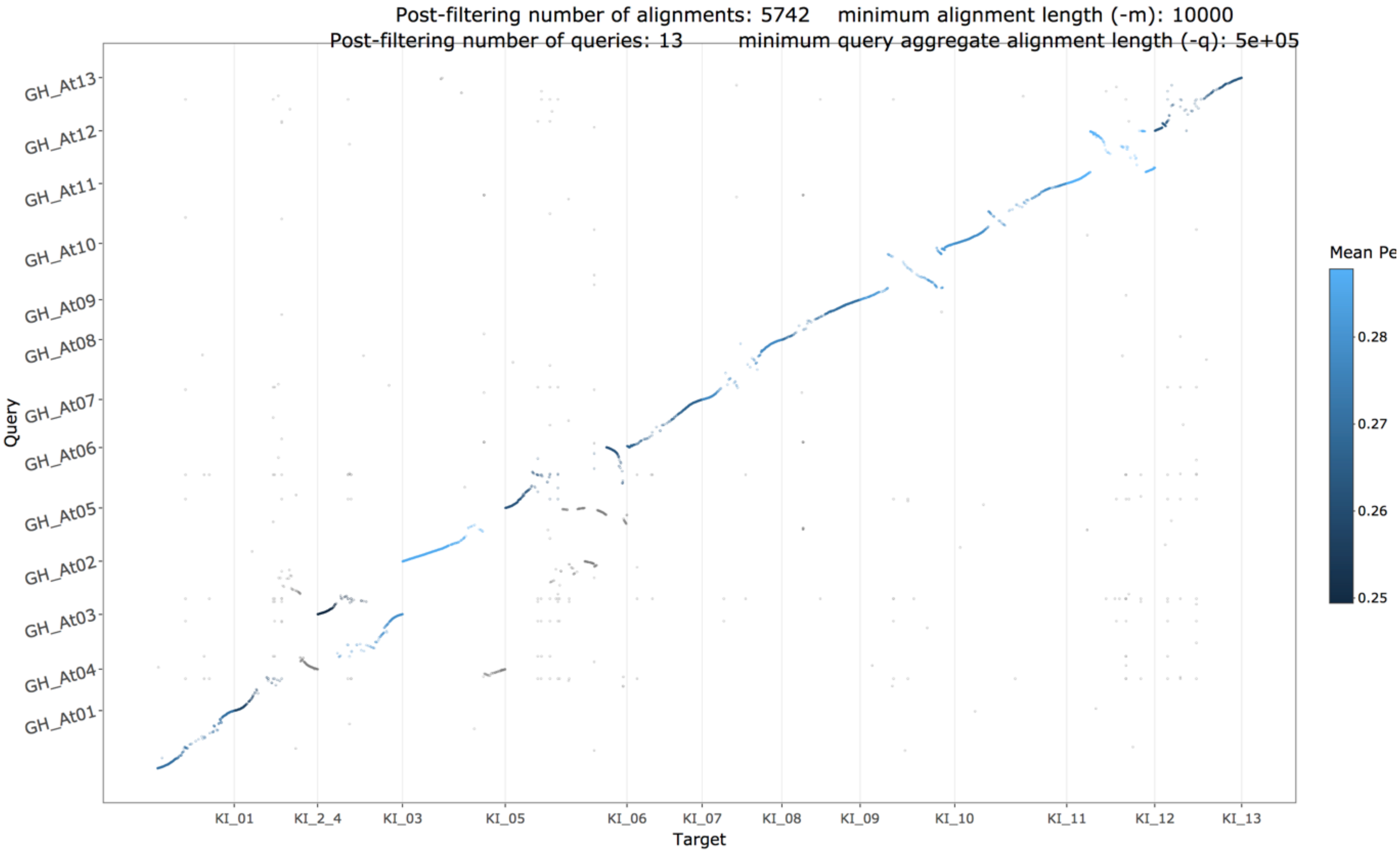

Fig 7. Dotplot of genome alignments between the genome sequence of *G. kirkii* and the genome sequence of D_T genome of *G. hirsutum*, where the 'T' indicates the A genome in the tetraploid nucleus).

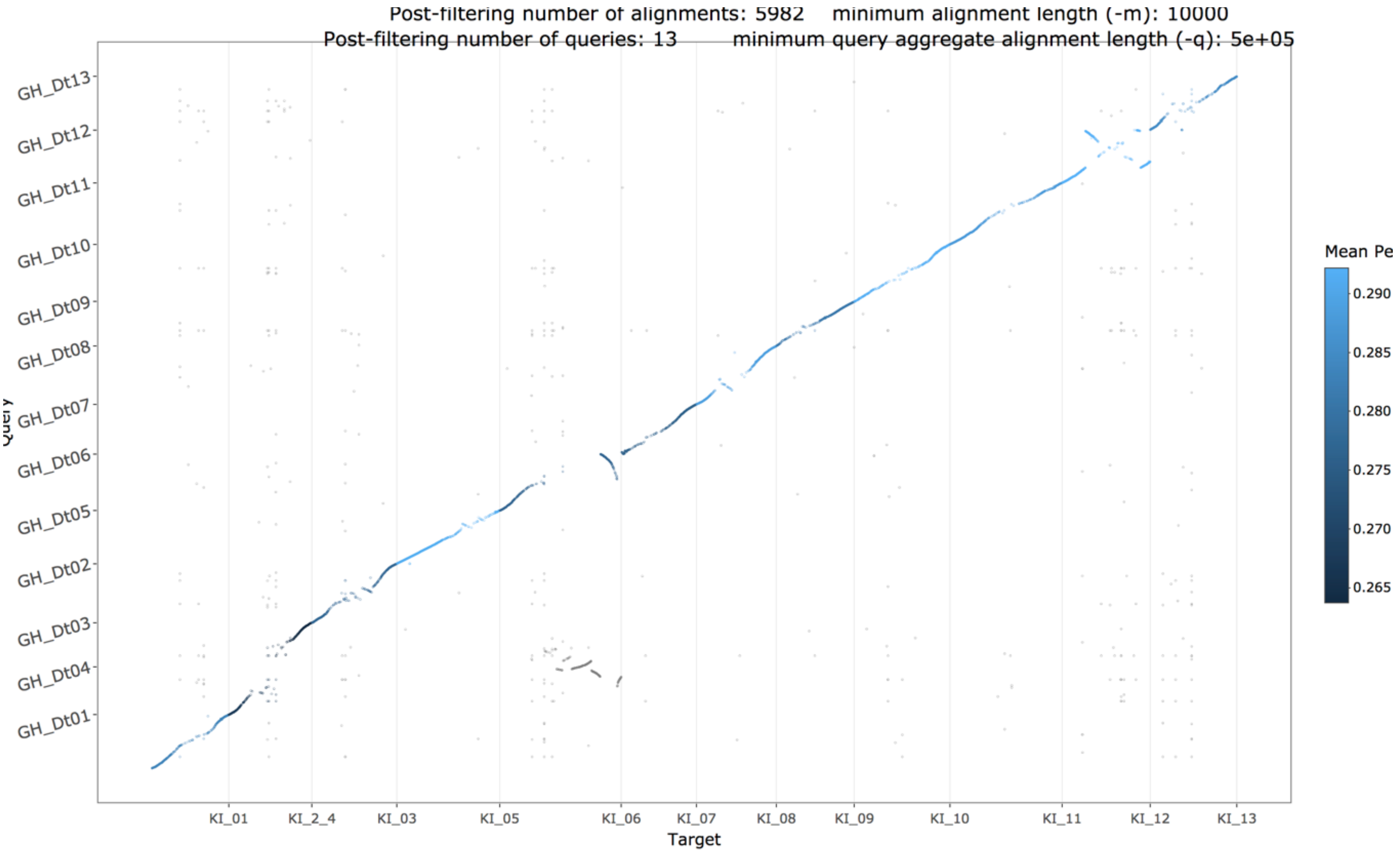

Fig. 8 Distribution of Ks values between coding sequences of intraspecific paralogs. The peak of Ks values between intraspecific paralogs suggests a genome-wide duplication event in the natural history of *Gossypioides kirkii.*

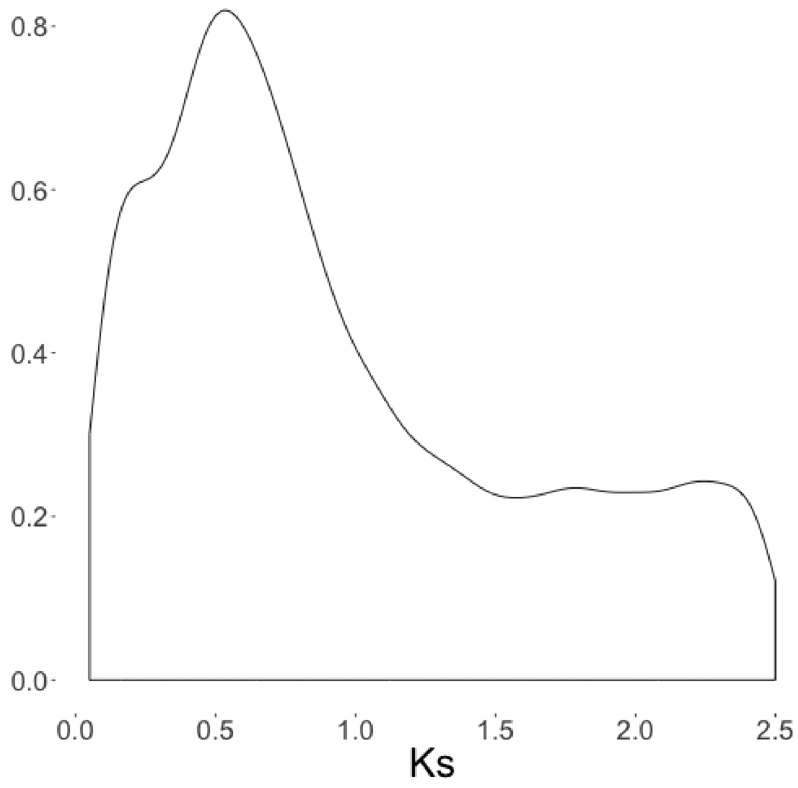

Fig. 9 A representative chromosome squash of *G. kirkii* demonstrating the lack of an exceptionally large chromosome, while n=12. A 35bp 7-mer telomeric oligo of TTTAGGG 5'-linked to alexafluor was used as a telomeric probe.

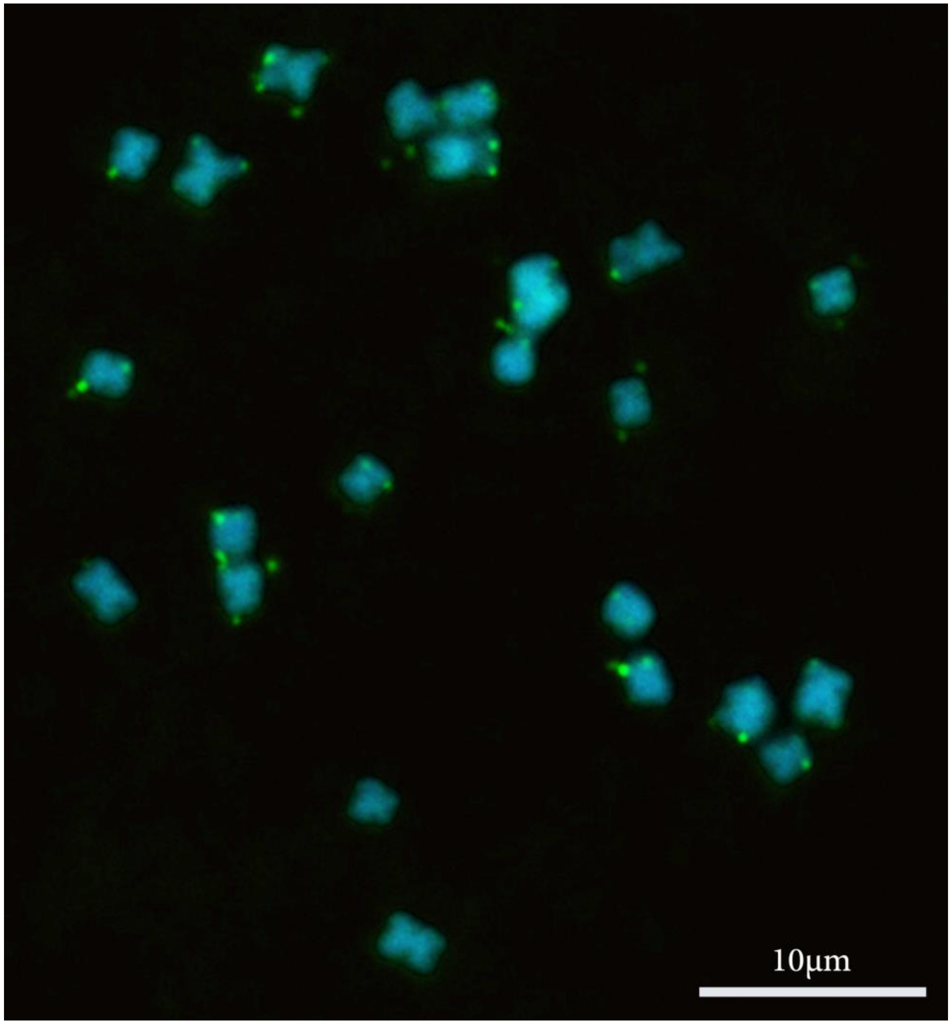

Fig. 10 *Kokia drynarioides* HiC reads mapped to the *Gossypioides kirkii* genome do not show any unusual interactions across the genome (in general), or in KI_06 and KI_2_4 (specifically). This suggests that these two chromosomes of *G. kirkii* have the same structure in the *K. drynarioides* genome.

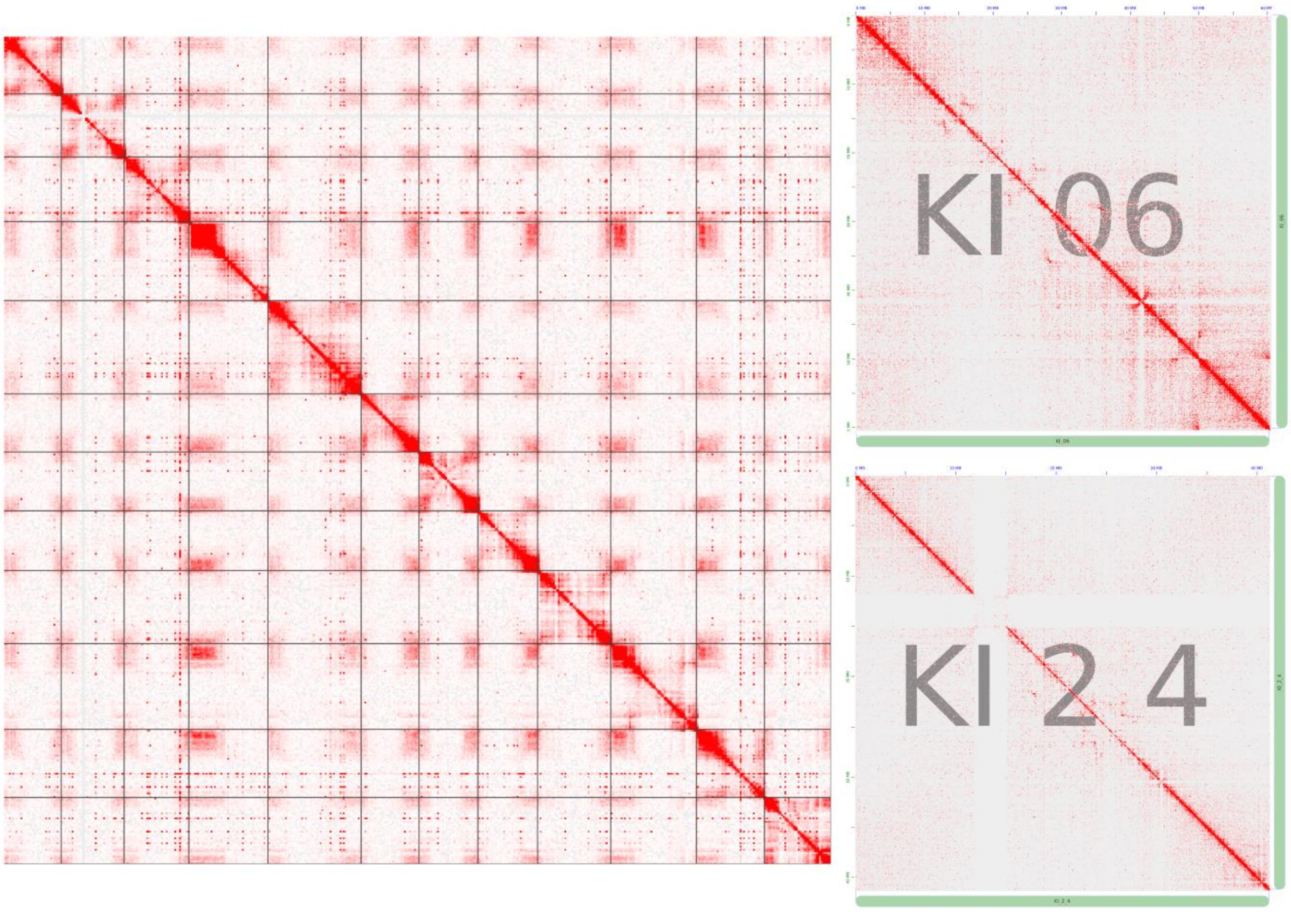

Table 1. PacBio sequencing of *Gossypioides kirkii*

Items	Raw reads	CANU corrected reads
Total number of reads	5,241,096	4,622,516
Total number of sequenced bases (Mbp)	40,300	39,999
Mean subreads length (bp)	7,689	na
N50 (subreads length, bp)	12,000	35,011
Coverage (X)*	6.85374E-05	68.02

*Coverage (x) = (read count * read length) / estimated genome size.

Table 2. Result metrics of Hi-C library sequencing

Datapoint	Value
Hi-C Read Length (bp)	150
Hi-C Read Pairs	255,901,392
Hi-C Read Pairs (%)	100.00%
Aligned Read Pairs	100,882,035
Aligned Read Pairs (%)	39.42%
0 MapQ Read Pairs	28,080,722
0 MapQ Read Pairs (%)	10.97%
Pairs aligned to same contig	39,834,952
Pairs aligned to same contig (%)	15.57%
Useful Hi-C Read Pairs	32,683,315
Useful Hi-C Read Pairs (%)	12.77%

Table 3 Results of Hi-C/JuiceBox scaffolding and repeat analysis.

				Gypsy		Copia		Everything TE		Gene # of homologous chromosomes				
Chr. ID	Contig number	Length of contigs	gene #	# LTR	% Chr.	# LTR	% Chr.	Cum. TE #	Cum. TE %	D-genome gene #	*G. kirkii* genes/D-genome genes	A-genome gene #	Chr (G#)	*G. kirkii* genes/A-genome genes
KI_01	11	38174213	2580	4892	23.8	574	1.5	8599	33	2768	0.93	2841	1	0.91
KI_2_4	65	41239026	2385	5953	27.8	610	1.3	10699	40.9	2689	0.89	1799	2	1.33
KI_03	21	42116415	2633	6251	26.3	604	1.4	10381	35.5	1875	1.40	2802	3	0.94
-	-	-	-	-	-	-	-	-	-	2943		2145	4	
KI_05	26	50980511	4296	5333	18.1	795	1.4	9750	26.5	2698	1.59	4310	5	1.00
KI_06	9	60359545	3972	7609	22.9	915	1.5	13404	32.1	2754	1.44	2560	6	1.55
KI_07	24	37252139	2615	4249	20.5	632	1.5	8146	30.2	3802	0.69	2709	7	0.97
KI_08	7	39602770	2824	4630	21.9	650	1.5	8658	31.3	2990	0.94	2981	8	0.95
KI_09	29	38744031	2717	4192	18.9	725	1.8	9065	29.2	4564	0.60	2840	9	0.96
KI_10	36	47257541	2823	6183	24.3	784	1.8	10535	34.2	2570	1.10	3092	10	0.91
KI_11	27	55708459	3979	7003	23	796	1.4	12384	32.6	2963	1.34	4131	11	0.96
KI_12	15	43711424	3072	6314	26.3	605	1.4	10122	34.5	1880	1.63	2917	12	1.05
KI_13	18	42971468	2784	6722	28.6	609	1.3	10761	37.6	2727	1.02	2845	13	0.98
TOTAL	288	538117542	36680	69331		8299				37223		37972		

Table 4 Assembly of the Bionano molecules

Metrics	Value
N Genome Maps	1287
Total Genome Map Len (Mb)	353.683
Avg. Genome Map Len (Mb)	0.275
Median Genome Map Len (Mb)	0.243
Genome Map n50 (Mb)	0.296
Total Ref Len (Mb)	488.537
Total Genome Map Len / Ref Len	0.724
N Genome Maps total align	1079 (0.84)
Total Aligned Len (Mb)	263.644
Total Aligned Len / Ref Len	0.54
Total Unique Aligned Len (Mb)	252.347
Total Unique Aligned Len / Ref Len	0.517

Table 5. Telomeres and centromeric regions identified on *Gossypioides kirkii* chromosome sequences (* denotes internal telomere sequences detected; L and R designate left and right telomere sequences).

Chrom.	Chrom. Len/(pos.)	Telomere L Sequence	Telomere L length	Telomere R Sequence	Telomere R length	Cent. pos. (MB)	Cent. Contigs (N)
KI_01	38174213	CCCCAAA	840 bp	GGGCTTC	650 bp	13-30	6
KI_01*	(1409359)	mixed	600 bp			-	-
KI_2_4	41239026	CCCGAAA	1640 bp	-	-	12-23	50
KI_03	42116415	CCCCGAA	1000 bp	GGGGTTT	240 bp	12-27	17
KI_05	50980511	CCCGAAA	630 bp	GGGGTTT	770 bp	25-45	2
KI_05*	(11666383)	CCCGAAA	1200 bp			-	-
KI_06	60359545	CCCTAAA	15100 bp	-	-	12-35	1
KI_06*	(39472230)	Ancient 4R	Ancient 4R	CCCTAAA	90bp	-	-
KI_06*	(49966020)	Ancient 6R	Ancient 6R	CCCTAAA	400bp	-	-
KI_07	37252139	CCCCGAA	500 bp	GGGGCTT	1000 bp	11-22	17
KI_08	39602770	CCCTAAA	24040 bp	GGGTTTA	1200 bp	8-25	4
KI_09	38744031	CCCTAAA	3700 bp	GGGGTTT	230 bp	7-22	27
KI_09*	(38725251)	Mixed	230 bp			-	-
KI_10	47257541	-	-	GGGGTTT	660 bp	11-26	1
KI_11	55708459	CCCGAAA	720 bp	GGGGTTT	550 bp	24-43	1
KI_12	43711424	CCCCGAG	1600 bp	TTTAGGG	250 bp	21-37	9
KI_13	42971468	-	-	GGGGTTT	950 bp	9-20	13

Table 6. Lengths and positions of rearranged segments (rows).

G. kirkii Genome						D-genome					
Chr	Start	Stop	Ancient Chr.	Length	Gene # Kirkii	Chr. (D-genome)	start	stop	Gene # D-genome	Chr. (A2-genome)	
KI_06	1	22,228,028	G6	22,228,027	1,327	Chr06	12,994	28,591,341	709	Chr06	
KI_06	22,228,029	28,064,089	G2	5,836,060	134	Chr02 (Chr02 & Chr13)**	4,979,849 & 22,998,639	6,847,873 & 30,096,801	183	Chr02 (Chr01)	77
KI_06	28,179,358	31,236,019	G4	3,056,661	117	Chr04 (Chr12)	2,305,581	3,638,538	99	Chr04	
KI_06	31,237,465	35,659,810	G2	4,422,345	120	Chr02 (Chr03)	7,289,667	12,893,851	146	Chr02	
KI_06	35,703,819	39,289,648	G4	3,585,829	193	Chr04 (Chr12)	1	2,287,262	191	Chr04	
KI_06	39,318,495	45,381,234	G2	6,062,739	458	Chr02 (Chr03)	1	7,259,363	503	Chr02	
KI_06	45,453,930	49,918,628	G4	4,464,698	368	Chr04 (Chr12)	3,641,499	10,485,129	418	Chr04	
KI_06	49,918,629	58,299,838	G6	8,381,209	1,007	Chr06	34,225,200	62,134,075	1,733	Chr06	
KI_06	58,299,839	60,288,420	G4*	1,988,581	235	Chr04 (Chr12)	20,888,526	10,542,282	275	Chr04	
KI_12***	1	11,536,898	G12	11,536,897	1,632	Chr12 (Chr08)	1	17,201,929	832	Chr12	
KI_12	11,569,140	35,545,665	G12	23,976,525	884	Chr12 (Chr08)	56,060,095	24,608,968	1,907	Chr12	1
KI_12	35,857,881	38,806,259	G12	2,948,378	120	Chr12 (Chr08)	57,250,888	56,102,745	138	Chr12	1
KI_12	38,882,059	43,628,311	G12	4,746,252	416	Chr12 (Chr08)	17,314,504	23,994,015	106	Chr12	

* Inversion of the end of KI_06
** Chromosomes in parenthesis are legacy names
***These K12 positions were determined by matching the D-genome. The A-genome match positions are similar locations, but exact numbers are not presented here.
